# Advanced magnetic resonance imaging in human placenta: insights into fetal growth restriction and congenital heart disease

**DOI:** 10.3389/fcvm.2024.1426593

**Published:** 2024-07-23

**Authors:** Eric Sadiku, Liqun Sun, Christopher K. Macgowan, Mike Seed, Janna L. Morrison

**Affiliations:** ^1^ Division of Cardiology, Department of Pediatrics, The Hospital for Sick Children, University of Toronto, Toronto, ON, Canada; ^2^ Department of Health Science, Queen University, Kingston, ON, Canada; ^3^ Translational Medicine Program, The Hospital for Sick Children, University of Toronto, Toronto, ON, Canada; ^4^ Department of Biophysics, Faculty of Medicine, University of Toronto, Toronto, ON, Canada; ^5^ Department of Physiology, Faculty of Medicine, University of Toronto, Toronto, ON, Canada; ^6^ Research Institute, The Hospital for Sick Children, University of Toronto, Toronto, ON, Canada; ^7^ Department of Diagnostic Imaging, The Hospital for Sick Children, University of Toronto, Toronto, ON, Canada; ^8^ Early Origins of Adult Health Research Group, Health and Biomedical Innovation, Clinical and Health Sciences, University of South Australia, Adelaide, SA, Australia

**Keywords:** fetal growth restriction, congenital heart disease, MRI, human placenta, BOLD, DWI, SWI, placenta oxygenation

## Abstract

Placental function plays a crucial role in fetal development, as it serves as the primary interface for delivery of nutrients and oxygen from the mother to fetus. Magnetic resonance imaging (MRI) has significantly improved our ability to visualize and understand the placenta's complex structure and function. This review provides an up-to-date examination of the most common and novel placental MRI techniques. It will also discuss the clinical applications of MRI in diagnosing and monitoring placental insufficiency, as well as its implications for fetal growth restriction (FGR) and congenital heart disease (CHD). Ongoing research using multi-parametric MRI techniques aims to develop novel biomarkers and uncover the relationships between placental parameters and pre-onset diseased states, ultimately contributing to better maternal and fetal health outcomes, which is essential to better guide clinical judgement.

## Introduction

The placenta is a transient, yet complex organ, involved in the proper growth and development of the fetus. The attachment of the uterine vessels to the basal plate of the placenta, controls the maternal-placental exchange of oxygen and nutrients (1). Similarly, the attachment of the umbilical vessels to the chorionic plate are responsible for placental-fetal transmission (1). Collectively, these vascular beds function through the chorionic villus, the functional unit of the placenta, which mediates the interaction between the uterine and umbilical vessels. Within the placenta, the uterine artery supplies maternal blood to the intervillous space, which surrounds the chorionic villus, and facilitates the transfer of oxygen and nutrients. The physiological relationship between structure and function is evident within placental anatomy, which fundamentally supports the maternal-fetal interchange.

Placental interactions between the maternal and fetal vasculature are essential in the efficient exchange of nutrients and gases to support fetal growth and development. The initial formation of the chorionic villi, representing the functional unit of the placenta, begins 13 days post-conception and is complete around 24–26 weeks, where mature intermediate villi (MIV) differentiate into terminal villi specialized in nutrient and gas exchange (2, 3). The placental vasculature closely parallels villi development, with the initial presence of fetoplacental capillaries at three weeks post-conception, followed by gradual capillary growth throughout the transition phase from immature intermediate villi (IIV) to MIV to terminal villi (3, 4). In particular, non-branching angiogenesis is a crucial process during the late-second to early-third trimester, occurring within the terminal villi, that minimizes the diffusion distance between maternal and fetal blood and further facilitates the growing needs of the fetus for oxygen and nutrients (3).

**Figure 1:**
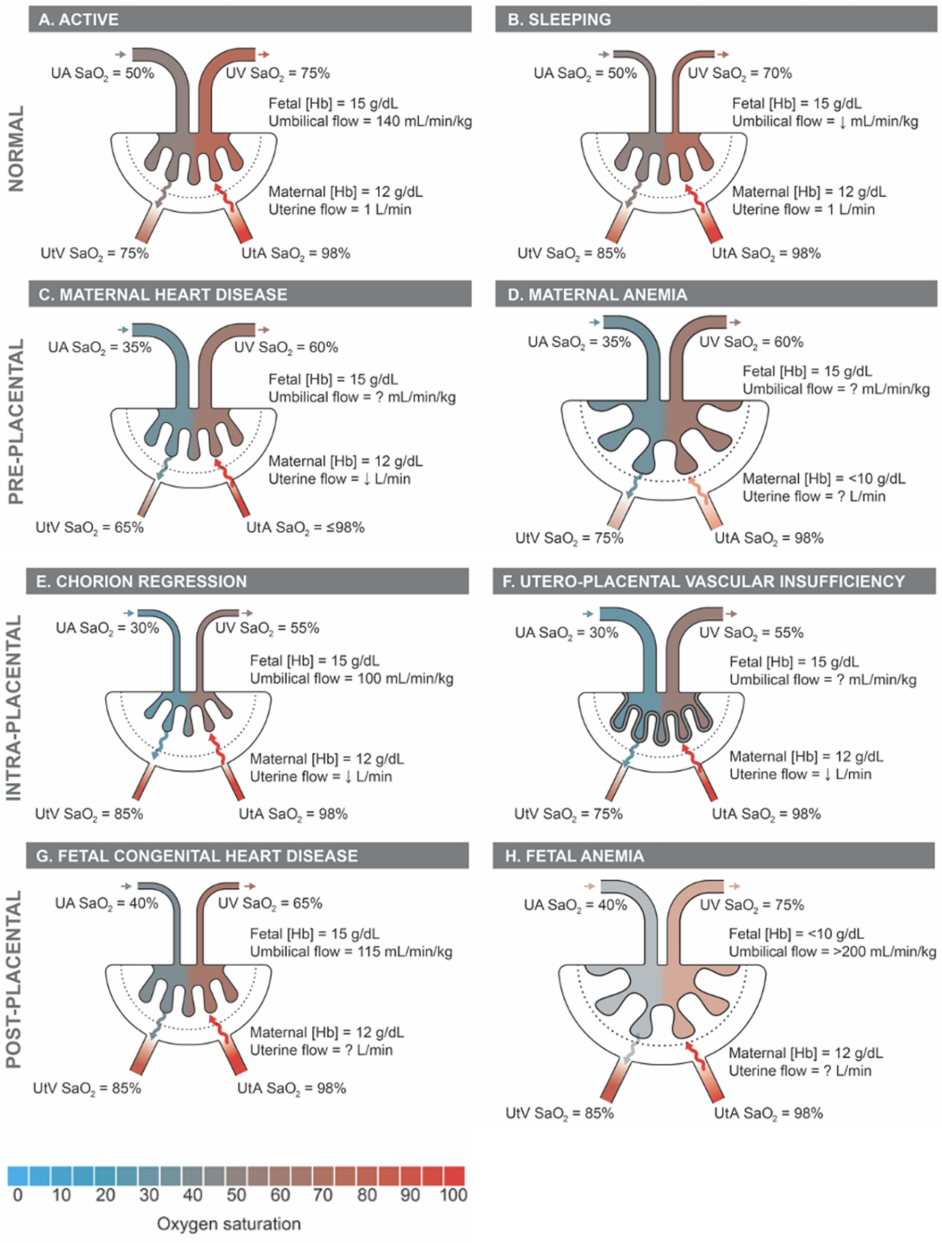
Proposed placental hemodynamics in normals and examples of pre-, intra-, and post-placental causes of impaired oxygen transport. Reference values from animal and human literature included where available with unknown values indicated as “?”. Q, flow; SaO_2_, oxygen saturation; Hb, hemoglobin concentration; UtA, uterine arteries; UtV, uterine veins; UA, umbilical arteries; UV, umbilical vein.

**Figure 2:**
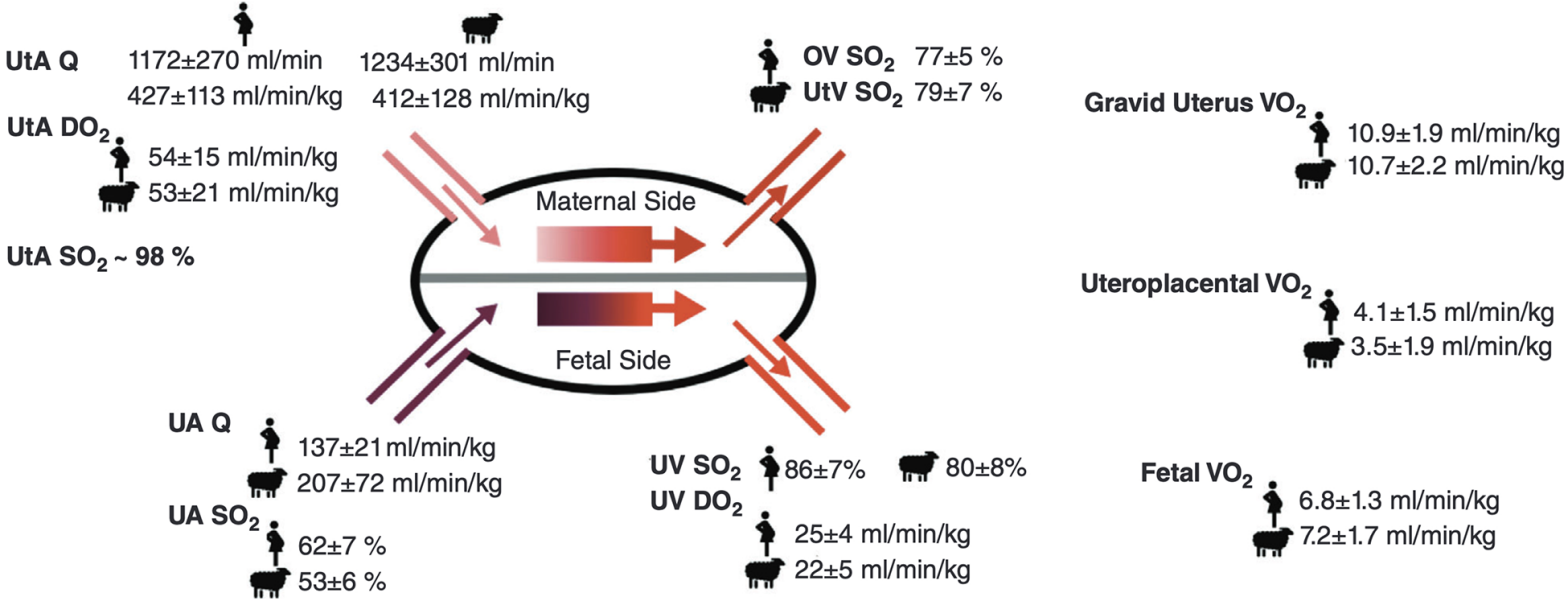
Placental hemodynamics, oxygen transport, and consumption in healthy normal human and sheep pregnant subjects in late gestation, Re-produced from Saini et al. (14) with permission from John Wiley and Sons. Q, blood flow; DO_2_, oxygen delivery; SO_2_, oxygen saturation; VO_2_, oxygen consumption; UtA, uterine artery; UtV, uterine vein; OV, ovarian vein; UA, umbilical artery; UV, umbilical vein; Hgb, [hemoglobin]; m, maternal; f, fetal.

Placental insufficiency occurs when there is a deviation from the normal processes of villous and vasculature development, leading to impaired transfer of nutrients and oxygen to the fetus. These physiological changes associated with placental insufficiency can lead to fetal chronic hypoxemia and/or hypoglycemic as well as FGR and CHD (5–8). Alongside placental insufficiency, issues involving dysfunctional implantation, such as placenta accreta spectrum and previa, or variant morpholgies, such as circumvallate placenta, may pose a risk to proper fetal development (9–11). The dependence of the fetus on maternal supply of nutrients and oxygen places a high demand for optimal placental function and effectively necessitates a lack of compromised blood flow. The placenta lacks neural innervation, which places a high reliance on hemodynamic forces and downstream vaso-regulatory mediator products, such as nitric oxide (NO), in facilitating proper development (8, 12). As such, variable hemodynamic forces caused by faulty fetoplacental vasculature formation may hinder necessary regulatory mechanisms to prevent FGR and CHD, among other developmental abnormalities (5, 8, 13). Figure 1 illustrates theoretical changes in placental hemodynamics that could occur in response to different maternal, placental, and fetal conditions, potentially leading to chronic fetal hypoxemia. These alterations can be detected using advanced magnetic resonance imaging (MRI) techniques, providing insights into the physiological adaptations and pathological processes affecting placental function. Saini et al., demonstrated the ability of MRI to measure blood flow and oxygen levels in the uterus and umbilical vessels, enabling the calculation of oxygen delivery to the pregnant uterus, placenta, and fetus (14) (Figure 2). Further study explored the feasibility of using MRI to assess maternal-fetal oxygen transport and consumption in conditions with altered uterine artery blood flow due to maternal position (15) or pharmacologic treatment (16). Relation to maternal positioning during healthy late-stage pregnancies has been applied to understanding the impact of maternal position or pharmacologic treatment to increase uterine artery blood flow on placental oxygen consumption (15–18). MRI offers a non-invasive way to gather detailed information about placental function and fetal well-being, which could aid in monitoring pregnancies and identifying potential complications (15).

Given the significance of fetoplacental vasculature development in the proper functioning and maintenance of the fetal environment, an update on the most current advances within the field of placental imaging is necessary. In this review, we will summarize the progress of various MRI technologies in assessing fetoplacental vascular composition and function, including levels of trans-placental perfusion and diffusion, as well as their abilities to measure oxygenation within the fetal blood (Table 1). This will be coupled with an analysis of the clinical implications of applicable MRI technologies on pregnancy complications associated with placental dysfunction such as FGR and CHD, involving the benefits and limitations of their use, as well as future directions.

**Table 1:** MRI methods used in human placenta imaging.

MRI method	What it measures	Sequence	Morphometry	Blood flow	Oxygenation (O_2_)	Contrast required	FGR	CHD	References
T2WI	Tissue Structure and pathology	Long time (LT), gradient recall echo (GRE) with low flip angle	Yes	No	No	No	T2* imaging scores were significantly lower in FGR placenta	Greater variance within placental lobular size in CHD patients	Chavhan et al. (19); Steinweg et al. (20); Wen et al. (21)
2D TOF MRA	Vasculature Structure	Conventional 2D/3D GRE	Minimal	Yes	No	No	–	–	Kuo et al. (22)
PC MRA	Vascular Structure + Flow	Balanced motion sensing bipolar gradients	Yes	Yes	No	No	–	–	Kuo et al. (22)
DCE-MRI	Vasculature Structure + Perfusion	T1-weigted gradient echo	No	Yes	No	Yes	–	–	Siauve et al. (23)
DWI + ADC	Diffusion + Microstructure	DWI with 2 b-values	No	Yes	No	No	Increased gestational age-induced decrease in ADC of FGR patients	–	Siauve et al. (23) Kuhnke et al. (24)
DWI + IVIM	Perfusion + Diffusion + Microstructure	DWI with 3 + b values	No	Yes	No	No	D and D* increased in late onset FGR *f* is decreased In late-onset FGR	–	(Siauve et al. (23); Kuhnke et al. (24); Andescavage et al. (25)
ASL	Perfusion	Flow sensitive alternating inversion recovery (FAIR)	No	Yes	No	No	–	VsASL indicates decreased global placental perfusion in second half og gestation within CHD patients Increased intra-placental perfusion variation compared to healthy controls	(Siauve et al. (23); Zun et al. (26)
DTI	Microstructure + Diffusion (unseparated)	Spin Echo—Echo Planar Imaging (SE-EPI)	No	No	No	No	–		Nana et al. (27)
DKI	Microstructure+ Diffusion (unseparated: non-gaussian distribution)	SE-EPI	No	No	No	No	Positive diffusion kurtosis values associated with normal placenta—deviations may indicate FGR	–	Zhu et al. (28)
BOLD	Oxygenation	T2* Relaxation times OR SE/gradient echo (GE)	No	No	Yes	No	Decreased placentaly oxygenation following maternal hyperoxic exposure in FGR patients	Higher placental oxygenation within single ventricle and aortic obstruction CHD patients, compared to healthy and other CHD patients	(Forster et al. (29)
SWI	Oxygenation	LE, fully flow compensated GRE	No	No	Yes	Yes—does not use extrinsic contrast agent	–		Barnes et al. (30)
NMR	Metabolic activity	Chemical shift imaging (CSI)	No	No	No	No	Increased placemtal glycine levels Altered placental uric acid cycle activity	–	Siauve et al. (23)

### Progression of placenta MRI

**Figure 3:**
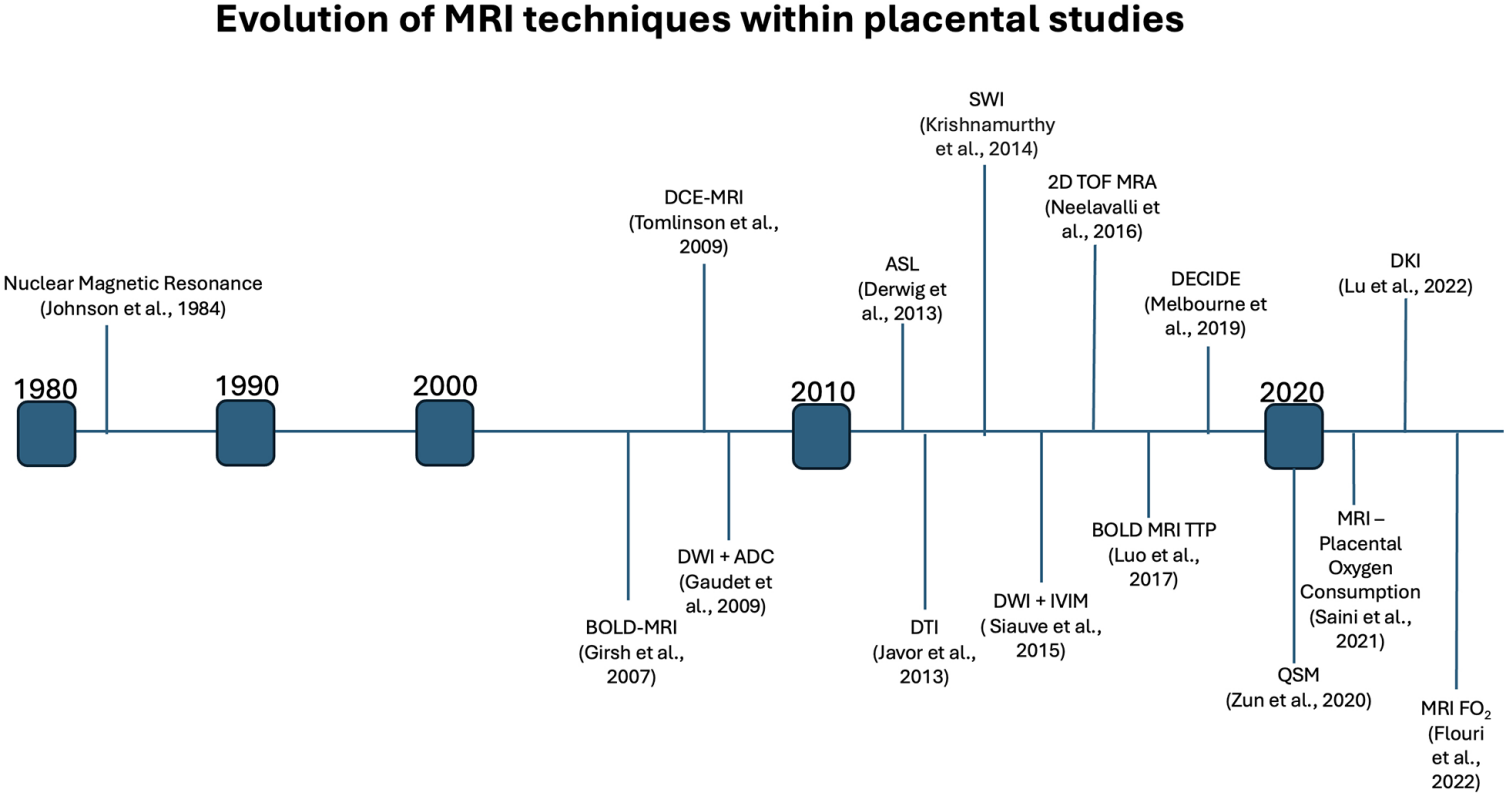
Timeline indicating the first noted usage of various MRI methods within placental studies, highlighting the extensive growth of placental MRI over the past decades.

MRI technology has come a long way since its inception in 1984, allowing a clear understanding of placental physiology. Placental imaging has presented unique challenges since its inception, often associated with the variability of placenta location, size, shape and vascular composition. Further, the need to maintain fetal safety limits the progression of the field. However, over the past four decades, researchers have developed increasingly complex MRI techniques (Figure 3), such as diffusion kurtosis imaging (DKI), which can measure non-normal water diffusivity within placental vasculature (31, 32). This parallels our growing understanding of the role that MRI may play in a better understanding of placental physiology and the impact of disease states on its structure and function.

### Current utility of MRI in the human placenta

#### Placenta structural and morphometric assessments

Although ultrasound is commonly used in pregnancy to measure placental and fetal growth and blood flow velocity in major vessels, it may offer a decreased ability for functional assessment, and limited viewing capabilities. The use of MRI offers an advantage in the antenatal study of the macrostructural and morphometric properties of the placenta (33). In particular, a broader viewing frame, along with greater contrast of soft tissue, and decreased operator-dependency provides benefits compared to ultrasound (33–35). Despite a slightly blurred boundary between the placenta and myometrium, the ability to clearly distinguish between the placenta and the amniotic fluid is significant and allows for precise measurements of placental shape and size at any gestational age (36, 37). The clinical relevance of placental morphometric properties is underscored—numerous studies have found correlations between placental weight and volume and postnatal properties such as birthweight, as well as health in later life (38–43). Furthermore, studies have indicated significant associations between properties such as placental weight, volume and surface area with placental insufficiency, fetal growth restriction (FGR) and Congenital Heart Disease (CHD) (44–47).

The use of T2-weighted imaging (T2WI) in MRI offers advantages for placental imaging compared to other techniques such as T1-weighted imaging (T1WI) (36). With T2WI, the normal placenta appears homogenous, particularly during the second trimester, providing a more effective baseline when compared to the heterogenous appearance with T1WI (36, 37, 48, 49). The transition to a more heterogenous appearance on T2WI, especially as the third trimester approaches, may indicate placental maturation—specifically, better-defined cotyledons with a more lobular appearance (48, 49). However, these observations should be integrated with additional structural and functional data to ensure a comprehensive understanding. Methods have been devised that combine 2D MRI images from axial, sagittal and coronal planes to construct a super-resolution 3D view of the placenta, enhancing segmentation and visualization capabilities (37, 50, 51). Super-resolution reconstruction methods aimed at improving the imaging of structural and morphometric properties hold significant potential to improve the diagnostic and analytic capabilities of placental MRI (37). Further, particular 3D-MRI models are capable of providing 3D views of the placenta, without the need for fusing 2D stacks (52). Usage of 3D-MRI models may decrease processing times, and improve image quality, due to decreased dependence on operator segmentation expertise.

#### Vascular structure and function

The success of many quantitative MRI techniques in placental imaging relies on the use of efficient vascular localizers to properly visualize the fetal, placental, and maternal vasculature (53). Time-of-flight (TOF) magnetic resonance angiography (MRA) represents a conventional, non-contrasting MRA approach that facilitates the ability to visualize vasculature in 3D (53). Ultrasound is a commonly used modality in clinical practice for imaging the fetal and placental vasculature due to its non-invasive nature, safety, and relatively low cost. However, ultrasound has limitations, particularly when visualizing deeper structures or those obscured by overlying tissues, bones, or gas. TOF-MRA offers an alternative approach that can overcome these limitations by acting as a supplemental measure to ultrasound results (54, 55). In the past, a study by Neelavalli et al. indicated the success of TOF-MRA in visualizing the fetal, placental and maternal vasculature when using 3.0 T MRI during the third trimester (53). While this study showed success in observing the vasculature of the fetus and the placenta, it was limited due to its testing parameters. For instance, the clinical reality of fetal imaging at that time relied on access to 1.5 T MRIs during earlier gestational periods. This was addressed by Qu et al., who conducted a study involving 2D TOF-MRA at 1.5 T and 3.0 T while also including an experimental group to determine the impact of gestational age. Regardless of 1.5 T or 3.0 T usage, the umbilical arteries were observed in all cases, and chorionic arteries were observed in most cases. Further, the repeated use of 3.0 T on three patients within the second and third trimesters revealed consistent images of the radial and spiral arteries, indicating feasible usage within the second trimester (55). This study led the way to the view that 1.5 and 3.0 T MRI can be used in fetal and placental imaging.

The limitations of TOF-MRA involve a bias towards higher-speed blood flow, which limits the resolution of less rapid blood vessels, as well as a clinical need for faster imaging times, which assists in combatting excessive fetal motion (53, 55). Future research should focus on improved resolution of lower-speed blood vessels using TOF-MRA techniques to address vascular localization concerns with modern usage.

Phase contrast (PC) MRA is a non-contrasting sequence that offers capabilities for the quantification and localization of blood velocity (37, 56). PC-MRA offers similar limitations as TOF-MRA, including long acquisition times; however, its 3D usage provides advantages over TOF-MRA, particularly related to enhanced detection of collateral flow or slower flow (57). PC-MRA is typically only used in cases where vascular structure and flow are essential, such as within the detection of stenosis (22). A study by Nii et al., which used 2D PC-MRA, emphasized its clinical utility by indicating a change in uterine blood in pregnant women, following administration of tadalafil, a phosphodiesterase-5 inhibitor, for treatment of FGR (58).

#### Placental perfusion

Dynamic contrast-enhanced (DCE) MRI is a valuable technique for imaging the placental vasculature, offering both visualization and quantitative analysis of microcirculatory parameters, through fast-imaging sequences that coincide with repeated administration of a contrasting agent (59). However, the use of gadolinium as a contrast agent in DCE-MRI during pregnancy has been associated with risks to the fetus due to its secretion into the amniotic fluid (60, 61). Some nonhuman studies have indicated low gadolinium concentrations within amniotic fluid following administration; however, human trials are necessary to confirm safe usage (62, 63). To address this issue, recent research has focused on developing alternate contrasting agents that are safer for use during pregnancy. One such alternative is a manganese-based contrast agent, Mn-PyC3A, which has been tested in non-human primates and found to be comparable in efficacy to gadolinium-based contrast agents (GBCAs) (64). Mn-PyC3A offers the advantage of dual excretion through both renal and hepatobiliary systems, reducing the risk of accumulation in the body (64). Further studies are needed to validate the long-term safety and sensitivity of Mn-PyC3A within various tissues and MRI techniques. An alternative, ferumoxytol, has been used safely in pregnant women for iron-deficient anemia and shows promise in recent studies using DCE-MRI with rhesus macaques (65–67). Prior studies have indicated that ferumoxytol has a safe administration profile through its lack of effect on placental and fetal growth and histopathology (68–70). DCE-MRI contributes significantly to assessing placental perfusion with studies demonstrating its ability to quantify maternal-placental blood flow in non-human primates and detect perfusion abnormalities in growth-restricted fetuses (71–73). A linear relationship between DCE-MRI perfusion metrics and gestational age suggests that this technique could be used to monitor proper fetal growth and identify developmental issues early (71). Recent research also highlights the potential of DCE-MRI for detailed spatiotemporal depiction of the placental lobes, offering new clinical perspectives for the early detection of abnormalities (74). However, the use of gadolinium as a contrast agent during pregnancy poses risks, promoting the exploration of safer alternatives like ferumoxytol for future applications in prenatal diagnostics.

Arterial Spin Labelling (ASL) represents a non-invasive MRI alternative to assess placental perfusion by comparing signal intensity within the placenta to blood travelling to the placenta (23). Common techniques within advanced ASL include spatial-selective labelling, which tags the blood based on location, and velocity-selective labelling, which tags the blood based on its velocity. Traditional ASLs, like pulse ASL (pASL) and continuous ASL (cASL), face limitations in accurate placental perfusion measurement due to inefficient labelling and signal loss (75). Pseudo-continuous (pcASL) and velocity-sensitive (vsASL) ASLs are advanced techniques that address some of these limitations, with pcASL offering improved signal-to-noise ratio (SNR) while relying on spatial-selective labelling (75–77). A study by Liu et al. used pcASL to demonstrate a relative decrease in placental lobule perfusion in high-risk pregnancies, indicating its clinical utility to identify placental dysfunction (78–80). Moreover, vsASL differs from the other ASLs through its replacement of spatial-selective labelling with velocity-selective labelling, which consequently prevents the measurement of arterial transit times (ATT) (80–83). This modification, alongside a decreased sensitivity to ATT, has allowed for a higher SNR in vsASL compared to pcASL (75, 84). Despite this, pcASL remains the clinical standard due to the novel nature of vsASL (75). The VESPA ASL technique combines pcASL and vsASL to optimize perfusion analysis while still measuring ATTs, offering a comprehensive approach to assessing placental health (83).

#### Placental microstructure

Diffusion-weighted imaging (DWI) is a non-invasive MRI technique that offers insight into placental microstructure and function by detecting the molecular motion of water protons (85). This method is particularly useful for identifying tissues with compromised membrane integrity in necrosis with placental failure (86). Consequently, DWI has clinical relevance in the early detection of changes related to various placental disorders, including placental insufficiency, FGR and CHD (25, 87–89).

Studies have utilized DWI to analyze placental health, with the apparent diffusion constant (ADC) and the intravoxel incoherent motion (IVIM) being key quantitative models for interpreting DWI data. The ADC reflects the overall mobility of water molecules within a tissue, while IVIM provides more detailed information about the microcirculation within the placenta. The ADC is a generalized measure of placental diffusion that cannot differentiate between true diffusion (D) and pseudo-diffusion (D*). Higher ADC values indicate less restriction for water movement and may suggest the presence of fluid-filled spaces, while lower ADC values indicate higher restrictions of water movement, typically in areas of high cellular density (90). ADC values have an inverse relationship with gestational age due to the normal maturation of the placenta during fetal growth (32, 50, 85). In growth-restricted fetuses, the gestational age decrease in ADC is larger and occurs earlier in gestation, offering an approach to distinguish between restricted and normal placental development (17, 50, 91–94). The D_mean_ and D*_minimum_ values derived from IVIM are also negatively correlated with gestational age (GA), which parallels the development of a fibrotic or calcified placental vasculature, and constitutes decreased diffusion and perfusion (32). The measurement of perfusion fraction (f) has revealed novel insight into placental dysfunction, with an inverse relationship between f and placenta dysfunction, which emphasizes the importance of perfusion within a healthy placenta (95, 96). Proper perfusion within the healthy placenta facilitates the transfer of oxygen and nutrients that are responsible for supporting normal fetal growth. This relationship allows for more effective identification of at risk of pregnancies, potentially enabling earlier intervention and improving outcomes. Anisotropic IVIM models account for the orientation of microvascular tissue and assume differences in results based on this (97). These models are indicated as a better fit for placental DWI and explain the diffusive properties more effectively than ADC modelling (97, 98). However, a limitation of the IVIM approach is increased total acquisition time, which can negatively impact patient compliance and may lead to motion artifacts (99).

DKI and the hybrid IVIM-DKI provide an enhanced view of placental health during pregnancy (32). These methods extend beyond traditional Diffusion Tensor Imaging (DTI) by accounting for the non-Gaussian distribution of water molecules within the placenta, offering a more detailed analysis of microstructural changes and tissue heterogeneity (100–102). However, recent validations have shown that DTI-based motion correction can effectively address misalignments in DWI caused by the movement of the fetus and mother, leading to more accurate quantification of placenta microstructures (103). Despite this clinical utility, DTI has limited biological specificity of various placental microstructural features (101). Within DKI, DK values are positively associated with gestational age, which indicates the potential of DKI in distinguishing cases of restricted fetal growth through low DK values (32).

#### Placenta oxygenation

Assessing placental oxygenation is a crucial aspect of monitoring placental and fetal development during pregnancy. The dynamic changes in oxygen within fetal and maternal placental vasculature across the three trimesters support the growth and development of the placenta and the fetus. In the first trimester, lower partial pressure of oxygen (PO_2_) around 20mmHg are physiologically normal and contribute to trophoblast proliferation (104, 105). As pregnancy progresses to the second trimester, there is an increase in PO_2_ to approximately 60 mmHg, which aids in trophoblast invasion and ensures proper placental development (104, 105). In the third trimester, PO_2_ begins to decrease and fluctuate, reaching an average of ∼40 mmHg. This decline correlates with the increasing oxygen demands of the growing fetus (104, 105). The importance of maintaining appropriate oxygenation is underscored by its strong association with conditions such as placental insufficiency, FGR, and CHD (106–109).

T1 and T2 mapping are common techniques associated with analysis of placental oxygenation and are utilized in a variety of signal sequences. T1 and T2 mapping is sensitive to oxygen saturation (SO_2_) levels, as well as hematocrit, which has led to its role in the evaluation of these measures (110). T1 and T2 relaxation times offer insight to placental function, and consequently may supplement current practices for determining placental function and corresponding fetal outcomes (95, 111). For instance, a study by Schabel et al., found a strong association between placental T2* and pregnancy outcomes within cases of placental insufficiency (111). Additional studies have indicated a predictive ability of T1 within cases of small for gestational age (SGA), which indicates its clinical potential (95). Further, the use of T1 and T2 in combination, allowed for significant distinctions between healthy- and FGR-pregnancies, compared to the isolated use of either (112).

**Figure 4:**
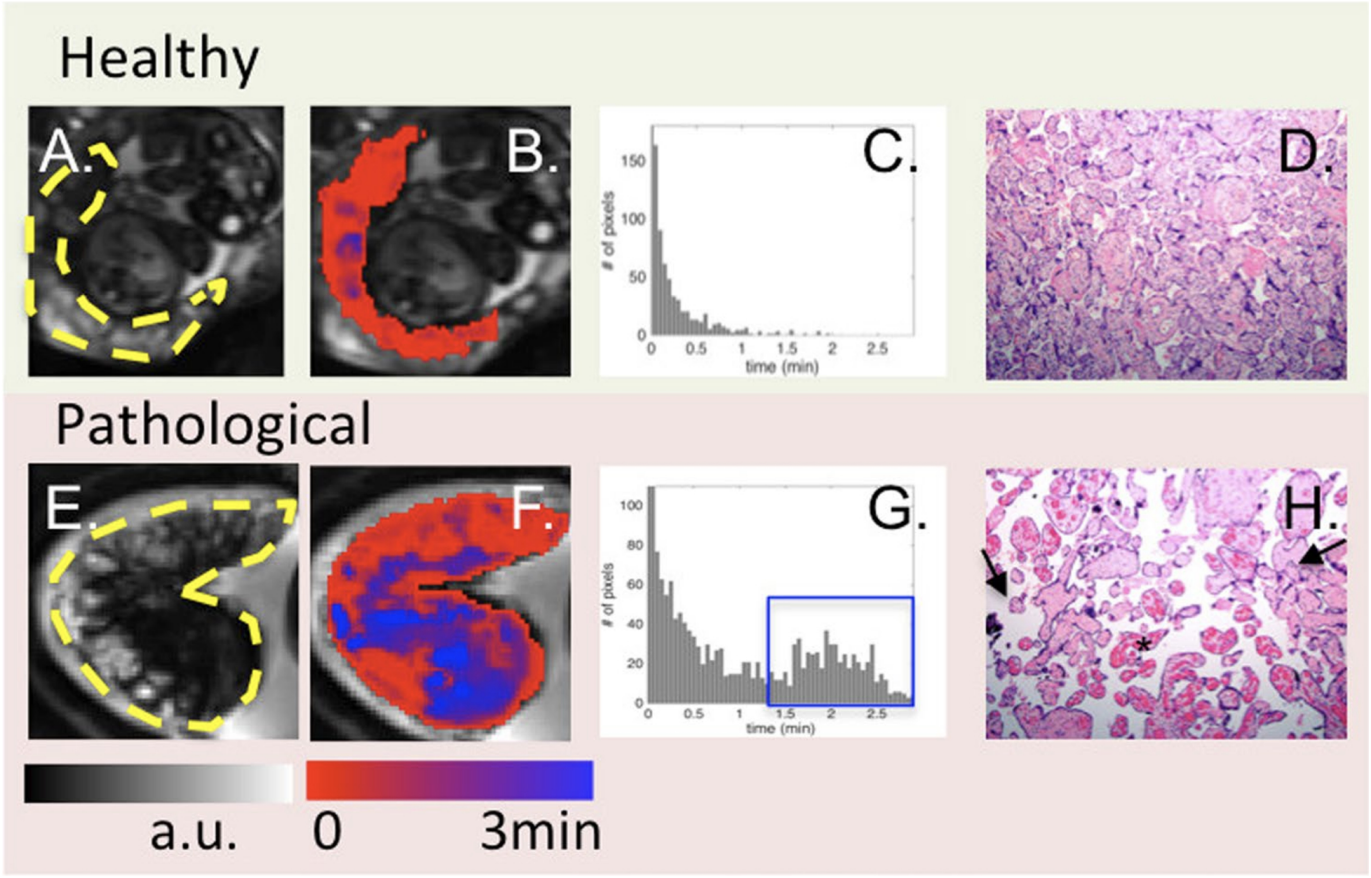
From left to right: BOLD images (**A**), time to peak (TTP) maps (**B**), histogram of TTP distribution (**C**) and histology (10X) (**D**). One control (top) is compared to one case with abnormal placental pathology (bottom). Yellow dashes in (**A, E)** outline the placenta. For healthy subjects, TTP values were short and placental histology was normal. For pathological cases, TTP values were longer and less uniform [blue regions in (**F**) and blue box in (**G**)]. Arrows in (**H**) point to the vascular villi, and the star identifies chorangiosis, re-produced from Luo et al. (114) used under CC BY 4.0.

Blood Oxygen Level Dependant (BOLD)-MRI is a sophisticated diagnostic tool that measures placenta oxygenation by detecting T2* relaxation time changes. This technique exploits the fact that deoxyhemoglobin, a component of red blood cells, has paramagnetic properties that affect the MRI signal intensity (113–115). Luo et al. used BOLD MRI to indirectly assess regional placental oxygen delivery, modelling the oxygenation response and generating time-to-plateau (TTP) maps (114). They found distinct TTP patterns in placentas with pathology vs. healthy ones, suggesting BOLD MRI could non-invasively detect placental dysfunction, complementing histopathological diagnosis (Figure 4). The R2* (1/T2*) parameter derived from BOLD-MRI scans is particularly important because it reflects the amount of deoxyhemoglobin present and indirectly measures placenta oxygenation: higher R2* values indicate lower oxygen levels.

Several studies have demonstrated that BOLD-MRI can quickly and directly observe changes in placental oxygenation in response to increased maternal oxygenation, whether achieved through supplemental oxygen or maternal breathing manipulation (107, 116). The rapid peak in ΔR2* values following oxygen administration underscores the sensitivity of BOLD-MRI in capturing real-time alterations in placenta function (107, 116). Furthermore, Saini et al. show that the maternal position during the BOLD-MRI scan influences placental oxygenation. These findings indicate that the supine position may impair oxygenation compared to the left lateral position (15), which supports standard clinical recommendations for the left lateral tilt position, aimed at optimizing blood flow between the mother and fetus (117). Additionally, Turk et al. point out the necessity of accounting for Braxton Hicks contractions within the interpretation of signal intensity due to their association with decreased global R2* levels within the placenta (118).

Another significant development in this field is the assessment of Placental Vascular Reactivity (PlVR), a novel and non-invasive index measured by BOLD-MRI that gauges the adaptability of the placental vasculature to changes in maternal oxygen levels. PlVR is primarily influenced by fluctuations in maternal CO_2_ levels, revealing a unidirectional relationship between mother and fetus regarding vascular responses (119). As an indicator of placental vascular integrity, PlVR quantification holds promise for clinical application due to its straightforward validation and use in a clinical setting (119). Collectively, this evidence portrays the significant clinical potential of BOLD-MRI in revolutionizing the real-time analyses of placental function and development.

Susceptibility Weighted Imaging (SWI) offers contrast capabilities by qualitatively interpreting the distortions in magnetic fields (86, 120). SWI is particularly sensitive to deoxygenated blood, which aids in distinguishing arteries from veins, making it valuable for oxygen saturation studies (121). Building on this, Quantitative Susceptibility Mapping (QSM) extends the capabilities of SWI by mapping the distribution of magnetic materials within tissues (120, 122), using both magnitude and phase data to achieve higher sensitivity to blood oxygenation compared to BOLD-MRI (123, 124). QSM has been used clinically to study the brain but has also shown potential in placenta research, where it has revealed positive correlations between spatial variation in oxygenation and gestational age. Effective use of QSM includes measuring baseline susceptibility, with amniotic fluid serving this role in placenta studies (122, 125).

The overlap of maternal and fetal blood within placental scans presents difficulties in functional analysis (93). Diffusion-Relaxation Combined Imaging for Detailed Placental Evaluation (DECIDE) is a novel multiparametric MRI technology that combines T2 relaxometry and DWI to compartmentalize the placenta into regions of maternal blood flow, fetal blood flow and placental tissue to address this issue (126). The clinical value of DECIDE has been validated in sheep studies and used to test efficacy of potential FGR therapies (16, 18, 50, 93). A study by Darby et al. used DECIDE to analyze the effects of tadalafil, on placental perfusion and found no change in SO_2_ of fetal blood in the placenta but an increase in maternal blood volume in the placenta, further supporting the clinical feasibility of DECIDE. DECIDE provides functional biomarkers capable of analyzing the SO_2_ of fetal blood in the placenta (FO_2_), which is directly approximated by T2 relaxation times (126, 127). Moreover, DECIDE detected decreased FO_2_ in the placentas of FGR patients and has indicated an inverse relationship with FGR severity (18, 91). Overall, DECIDE FO_2_ offers significant potential in differentiating FGR from small but normoxemic fetuses, which offers precise predictions of fetal growth up to three weeks in advance and offers significant potential for early diagnosis within FGR patients (93).

#### Placenta metabolism

The study of placental metabolomics represents an emerging field of research that has the potential to enhance our understanding of biomarkers and pathogenesis related to placenta disease (128). This is a burgeoning area with potential for deepening our comprehension of conditions such as FGR and CHD (129–131). Nuclear Magnetic Resonance (NMR) is a key analytical tool in this domain, enabling the real-time and *in vivo* examination of placental metabolites (128). Within the scope of imaging technologies, NMR may also be referred to as MR spectroscopy (MRS) (94). Proton MRS (H-MRS), a common metabolomic technique, has been instrumental in detecting disturbances in metabolite concentrations and biochemical pathway activities within the placenta (129, 132). Deuterium MRS (D-MRS), a non-invasive alternative to H-NMR, holds promise for clinical applications due to deuterium's inertness in metabolism and non-radioactive nature (133, 134). Studies using D-MRS have identified increased lactate in preeclamptic placentas, suggesting heightened glycolysis and hypoxic stress (134). High-resolution magic angle spinning (HR-MAS) MRS has emerged as an *ex vivo* method capable of measuring tricarboxylic acid (TCA) cycle placental metabolic analysis. Using this method, the HR-MAS MRS could identify and quantify various TCA cycle intermediates in placentas from mothers with hypercortisolemia (135, 136). Building on HR-MAS MRS, Comprehensive Multiphase (CMP) MRS enhances the differentiation of various phases within complex tissues like the placenta, offering detailed characterization that can improve the detection of abnormalities (137, 138). Clinical applicability of CMP MRS was demonstrated through the identification of altered amino acid concentrations in preterm birth placentas, underscoring its value in elucidating metabolic changes associated with placental pathologies (138, 139).

### Clinical implications in FGR and CHD

FGR occurs in ∼10% of pregnancies and is associated with poor outcomes including an increased risk of stillbirth, premature birth and admission to the neonatal intensive care unit. Furthermore, there is an association between poor growth *in utero* and the risk of non-communicable chronic disease in adulthood (140–142). Although FGR has maternal, placental and fetal causes, each generally results in reduced substrate (oxygen and/or nutrients) delivery to the fetus such that the fetus does not reach its genetic growth potential and has a birth weight less than the 10th centile, although Delphi consensus reports suggest the 3rd centile is most clinically relevant (143). The fetus mounts a hemodynamic and endocrine response to reduced substrates that results in changes in the structural and functional development of most organ systems (6, 144). These responses are the basis of fetal programming of increased risk of hypertension, coronary artery disease, diabetes, and obesity in adulthood.

Strikingly, nearly 50% of all FGR fetuses go undetected until after birth (106), despite improvements in obstetric imaging and management (145). Distinguishing SGA from FGR can be difficult (146). Thus, clinical decisions about when to deliver the FGR baby may not be optimal (145). To avoid stillbirth, for example, many FGR/SGA babies are delivered preterm and may face poor outcomes associated with immature organs (147).

Thus, early detection of placental dysfunction to allow proper clinical care is essential (148–150). Despite the lack of a gold-standard definition for FGR diagnosis, improved early detection and fetal monitoring are within the scope of modern MRI techniques (151). Even when FGR is identified, suboptimal monitoring protocols hinder the precise determination of the most advantageous timing for intervention (106, 148). Innovations within MRI techniques have facilitated their use for assessing placental macro- and microstructure, as well as placental oxygenation and metabolism (14, 23, 25, 32, 53, 59, 71, 85, 94, 115, 120, 129, 132, 152). Studies on placental morphometric characteristics and vascular structure have indicated significant relationships involving decreased placental volume (44). This is coupled with studies showing increased D and D* levels, with decreased f signals in late-onset FGR patients (25). These novel biomarkers of FGR may assist in guiding clinical judgements regarding proper care practices. Further, studies using BOLD-MRI have identified decreased placental oxygenation in the placentas of fetuses with FGR following periods of maternal hyperoxygenation (106, 107). This underscores the clinical relevance of oxygenation within more effective FGR care. Moreover, studies involving H-NMR have indicated elevated glycine levels and altered urea cycle activity within FGR patients, highlighting the prevalence of metabolic shifts (129). The future of MRI within FGR patients will likely involve multi-parametric and multi-compartment MRI techniques to allow for more optimal diagnostic and monitoring abilities. For instance, a study by Aughwane et al. used DECIDE, to not only indicate decreased placental oxygenation within FGR patients but also account for differences across varying gestational ages to produce more accurate parameters (91). Collectively, the use of various MRI technologies has provided more specific parameters and physiological biomarkers that can significantly advance the detection and monitoring of FGR, which may decrease the prevalence of stillbirths and faulty postnatal development within these patients.

CHD has been increasing in incidence over the last two decades (153, 154), and its diagnosis and management has been improved through MRI analysis (155–159), and the assessment of placental function. This variation in case incidence could reflect changes in diagnostic protocols for CHD or advancements in medical diagnostic capabilities; however, it highlights the need for improving clinical diagnostic and monitoring technologies. The development of the fetal heart is closely related to the development of the placenta, which emphasizes the importance of placental imaging (5, 160, 161). Consequently, CHD diagnosis and management may be more optimally approached through MRI analysis of placental structure and oxygenation (45, 47, 89, 108, 109). A study by Steinweg et al. used a T2*-based MRI to find that fetuses with CHD displayed greater variance within placental lobular size compared to healthy controls (20). Similarly, placental volume exhibits a positive relation with fetal birth weight; however, within CHD, placental volume displays a steeper positive relation with birth weight, which may indicate a compensation effect in the placenta (162). These structural observations may offer enhanced phenotypical indications of CHD, which may support diagnostic and monitoring efforts. Further, a study by You et al. used BOLD-MRI to demonstrate a relatively higher increase in placental oxygenation within single ventricle (SV) and aortic obstruction CHD compared to healthy controls and other CHD patients (116). In particular, this finding offers potential therapy for improving placental oxygenation within SV and aortic obstruction CHD and highlights the need to differentiate between various types of CHD. Various types of CHD exist, each with unique compensatory mechanisms that add to the clinical complexity of managing these diseases (163). MRI usage within CHD appears limited due to the lack of studies examining placental metabolism as a physiological biomarker, but its clinical implementation offers benefits and would function effectively as a supplement to current clinical protocols based on its ability to provide an understanding of placental function.

### Challenges and future directions

The challenges associated with placental MRI largely stem from patient-based limitations, particularly those related to excessive fetal and maternal motion (48, 164). This motion can lead to signal acquisition issues and result in image degradation (165, 166). Typical examples of this non-rigid motion involve placental deformation, maternal respiration patterns, uncontrolled uterine contractions and fetal body and breathing movements (34, 118). Techniques like Deformable slice-to-volume registration (DSVR) have been developed to correct misaligned or degraded images. However, it presents significant limitations involving improper handling of severe motion-induced degradations and automatic rejection of outliers, which may exclude useful information (167). To address these concerns, researchers are exploring the use of artificial intelligence (AI) for automatic segmentation of the placenta (168, 169), which could reduce the time-consuming and complex nature of manual segmentation practices (169).

Another issue is the presence of tissue interfaces within the placental environment, which can further degrade signals and images. The transition to 3 T MRI has made this a growing concern, as it increases image distortion compared to 1.5 T MRIs. Concerns including extreme acoustic effects and prolonged exposure to 3 T signals have been cited as potential safety hazards; however, some of these have been addressed (170, 171). Previously, 3 T was believed to be harmful to fetal development, but recent studies have found it safe for use within the fetal environment without demonstrating an excessive specific absorption rate (170).

To promote standardization in placental MRIs, implementing a validated scoring system should be a goal, as this could improve inter-observer reproducibility. Studies have shown that using MRI scoring systems can increase predictive values in determining future outcomes, offering strong clinical potential for their use in placental MRIs (172, 173).

## Conclusion

Evolving placental MRI technology now enables earlier detection and management of fetal conditions, featuring improved imaging clarity, minimized motion interference, and an array of diagnostic biomarkers. These advances aim to predict pre-dysfunction disease onset, enhancing therapeutic efficacy across gestational stages. Future directions focus on refining protocols and discovering new biomarkers to integrate placental MRI as a standard in clinical practice for optimal perinatal health.
